# First report of *Lasiodiplodia* *euphorbiaceicola* and *Neoscytalidium* *dimidiatum* causing dieback on *Cattleya* spp. (Orchidaceae) in Brazil

**DOI:** 10.1007/s42770-026-01935-7

**Published:** 2026-04-20

**Authors:** Pedro Thiago Santos Nogueira, Danilo Oliveira Ramos, Camila Santana de Oliveira, Jadson Diogo Pereira Bezerra, Olinto Liparini Pereira

**Affiliations:** 1 Programa de Pós-Graduação em Fitopatologia, Departamento de Fitopatologia, Universidade Federal de Viçosa, Viçosa, Minas Gerais 36570-900 Brazil; 2 Programa de Pós‑Graduação em Biologia da Relação Parasito‑Hospedeiro (PPGBRPH), Instituto de Patologia Tropical e Saúde Pública, Universidade Federal de Goiás, Rua 235, S/N, Setor Universitário, Goiânia, Goiás CEP 74605‑050 Brazil; 3 Laboratório de Micologia (LabMicol), Departamento de Biociências E Tecnologia, Instituto de Patologia Tropical e Saúde Pública, Universidade Federal de Goiás, Rua 235, S/N, Setor Universitário, Goiânia, Goiás CEP 74605‑050 Brazil

**Keywords:** *Botryosphaeriaceae*, Etiology, Plant pathogen, Orchid

## Abstract

The orchid trade is a significant global industry, with annual sales of approximately USD 2 billion. Orchids rank among the most important potted plants sold in Brazil. *Cattleya* is one of the most widely cultivated Brazilian orchid genera, with some species threatened with extinction. Despite their economic relevance, the etiology of many orchid diseases remains unknown. One of these diseases is the pseudobulb dieback observed in *Cattleya* species in orchid nurseries in Goiânia municipality, Goiás state in Brazil. Symptomatic plants of *C. amethystoglossa*, *C. guttata*, and *C. nobilior* exhibited necrosis at the pseudobulb apex, premature leaf drop, and downward lesion progression. Isolates were morphologically identified as belonging to *Neoscytalidium* (*Botryosphaeriaceae*) and *Lasiodiplodia* (*Botryosphaeriaceae*). To confirm the disease etiology, pathogenicity tests were performed with each isolate. Multilocus phylogenetic analysis identified four isolates as *Neoscytalidium dimidiatum* (from *C. amethystoglossa*), *N. dimidiatum* (from *C. guttata*) and two isolates of *Lasiodiplodia euphorbiaceicola* (from *C. nobilior)*. All isolates induced dieback symptoms, and reisolations confirmed pathogenicity, fulfilling Koch’s postulates. This is the first report of *L. euphorbiaceicola* worldwide and *N. dimidiatum* in Brazil causing dieback disease in *Cattleya* spp.

## Introduction

Orchids are adapted to various edaphoclimatic conditions, but the greatest diversity occurs in the tropics [1]. The Neotropics are the region with the greatest orchid diversity [2], and approximately 205 genera and 2,650 species recorded for the Brazilian flora, of which about 1,800 are endemic, with the Atlantic Forest being the richest biome in the country [3, 4].

Brazilian *Cattleya* has a wide distribution, with the country having the greatest diversity of the genus, with more than 80% of the species being endemic [5, 6]. The genus is also of economic importance, as it is one of the main commercial orchid genera, with species and hybrids producing flowers of admirable size, shape, colour, and fragrance [7–9].

Within the Brazilian context, orchids are among the most cultivated potted plants, along with lilies [10], and although the country has great potential in ornamental production, the commercialization of orchids is modest, corresponding to approximately BRL 10 million (ca. USD 2 million) annually [11]. Globally, these ornamentals of various sizes, shapes, and colours hold a prominent position in the market, both as cut flowers and potted plants. Additionally, orchids also have great importance in the cosmetics and pharmaceutical industries [12]. In 2000, the global demand was estimated at approximately 1.6 billion orchids, corresponding to over USD 2 billion [13].

Along with the increase in global orchid trade and the popularization of cultivation, there has been an increase in the incidence of diseases in these plants [14]. Various fungal diseases can cause damage to production, either through plant loss or product depreciation, the most common being: stem rot (*Fusarium* spp.) [14], rust (*Sphenospora kevorkianii*) [15], anthracnose (*Colletotrichum karstii*) [16], black rot (*Phythophthora heterospora*) [17], and leaf spots [18, 19]. However, there are significant gaps in our understanding of the etiology of fungal diseases in orchids.

Plants of *Cattleya amethystoglossa*, *C. guttata*, and *C. nobilior* from a commercial orchid nursery were observed with typical dieback symptoms, with black to light brown necrosis at the top of the pseudobulb, followed by early leaf drop and downward progression of the necrosis. To date, there are no reports of dieback affecting orchids in the country, and consequently the pathogen associated with this disease remains unknown. A detailed study of the etiology of the disease is a fundamental step for effective pathogen control, given the great diversity of orchids and the considerable market for these plants.

## Materials and methods

### Sample collection and isolation

**Fig. 1:**
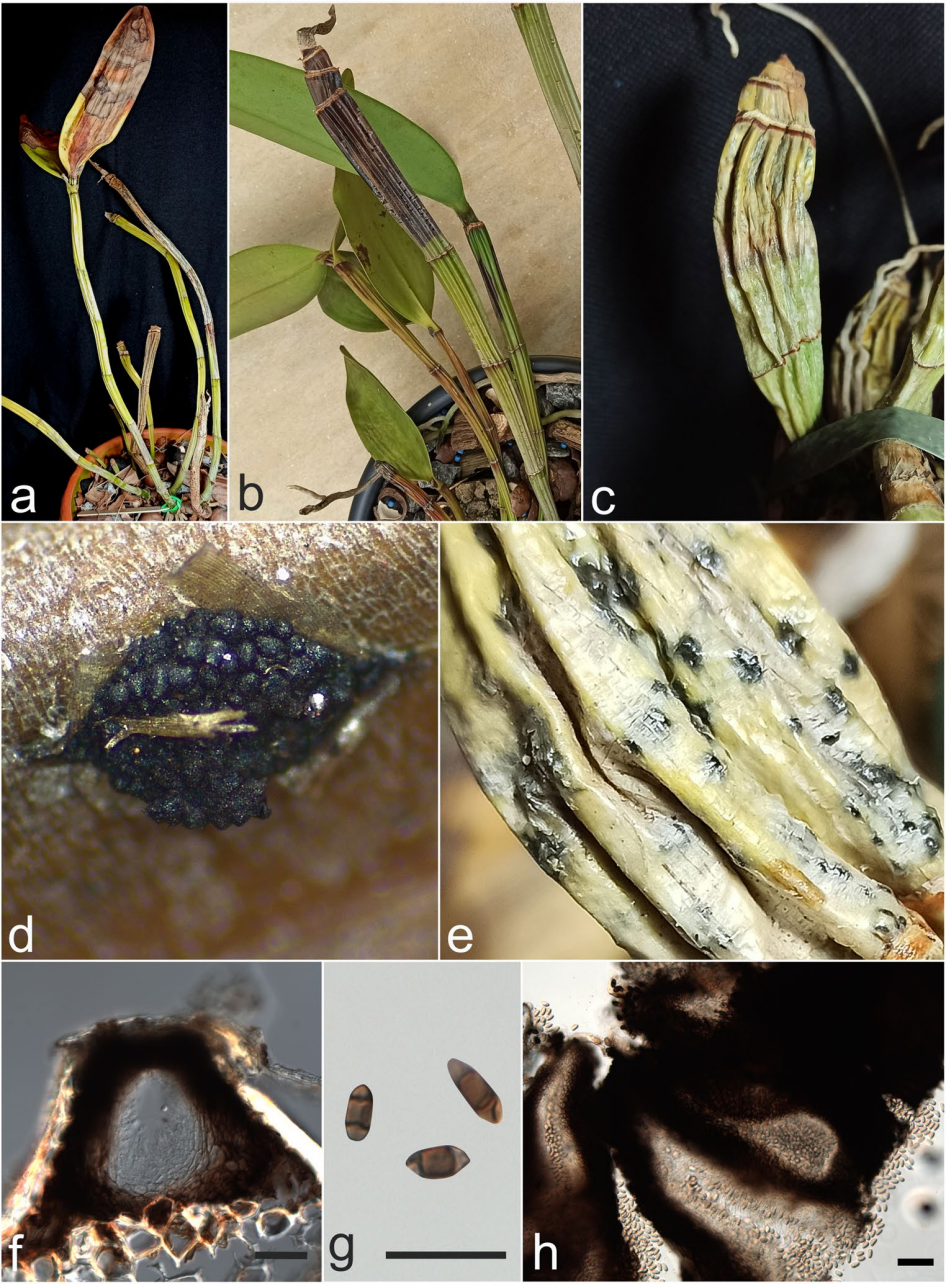
Symptoms and signs of dieback observed naturally in *Cattleya* spp. **a** – **c** Dieback symptoms in *C. amethystoglossa*, *C. guttata* and *C. nobilior*, respectively. **d** Pycnidium of *Neoscytalidium dimidiatum* in *C. amethystoglossa*. **e** Pycnidium of *Lasiodiplodia euphorbiaceicola* in *C. nobilior*. **f** Section of a pycnidium of *N. dimidiatum* in *C. guttata.*. **g** *N. dimidiatum* pycnidial conidia in *C. amethystoglossa.* **h** Section of a pycnidium of *L. euphorbiaceicola* in *C. nobilior*. Scale bars f–g = 20 μm, h = 100 μm

Symptomatic plants of *Cattleya* spp. were sampled in 2022 and 2023 from a commercial orchid nursery in Goiânia municipality, Goiás state, Brazil (Fig. 1). Approximately 70% of *C. amethystoglossa* and *C. guttata* orchids showed dieback symptoms, culminating in the death of these plants. *Cattleya nobilior* had fewer symptomatic plants, with 40% of the plants affected by the disease. Samples were transported to the Laboratório de Micologia e Etiologia de Doenças Fúngicas de Plantas (UFV) in Viçosa or to the Laboratório de Micologia (LabMicol-IPTSP-UFG) in Goiânia. Monosporic isolates were cultured on potato dextrose agar (PDA) at 25 °C under a 12-hour photoperiod for 7 days.

The isolates were stored in sterile distilled water, in silica gel at 5 °C, and in microtubes containing 10% glycerol solution and deposited in the collection “Coleção Otávio Almeida Drumond” (COAD), housed at the Universidade Federal de Viçosa, Minas Gerais, Brazil.

### DNA extraction, sequencing and phylogeny

Total DNA was extracted from fungal mycelium grown on Petri dishes containing PDA medium for 3 days at 25 °C in the dark, using the Promega Genomic DNA Purification Kit (‘Wizard Genomic DNA Purification Kit’), as described by [20].

The target sequences of the Internal Transcribed Spacer (ITS), β-tubulin (*TUB*)and translation elongation factor 1-α (*TEF1*-α) were amplified using the primers ITS1 and ITS4 [21], Bt2a and Bt2b [22] and EF1-728 F and EF2 [23, 24]. The annealing temperature was 52 °C for all primers combinations. The integrity and size of the PCR fragments were verified using agarose gel electrophoresis before sequencing.

The PCR products were purified and sequenced by Macrogen Inc, South Korea. The nucleotide sequences were edited with the FinchTV v.1.4.0 software (GEOSPIZA Inc.). All sequences were manually verified, and nucleotides in ambiguous positions were clarified using the primer sequences in both directions. The new sequences were deposited in GenBank. Sequences of pathogen species were obtained from GenBank for isolate identification.

For phylogenetic analysis, the sequences were compared with GenBank using megaBLAST tool. Sequences with high similarity with our isolates were obtained from the GenBank to create a database of *Botryosphaeriaceae*. The closest sequences were aligned using MAFFT [25], manual modifications were made using MEGA v. 7 software [26]. The resulting alignment was deposited in Figshare (10.6084/m9.figshare.c.7987916). Maximum Likelihood (ML) was performed using IQ-TREE 2.2.2.7 [27] following the parameters described by [28]. The tree was visualized in FigTree [29] and exported to graphic programs.

### Morphology of fungal isolates

A representative isolate from each clade identified in the phylogenetic analysis was used for morphological characterization. The isolates were subcultured onto plates containing water agar 2% (WA) medium with *Pinus* needles. The plates were incubated at 25 °C under a 12-hour near-ultraviolet light photoperiod (wavelength range: 300–400 nm) for the formation of reproductive structures [30]. Sections of fungal structures were made manually and placed on slides containing lactoglycerol for visualization under a light microscope (Olympus CX 31). Images were obtained using an Olympus BX 53 light microscope equipped with a digital camera (OLYMPUS Q-Color5 ™). Thirty measurements of all relevant morphological characters were made using the cellSens Dimension 1.9 software (OLYMPUS) to confirm species identification.

### Pathogenicity test

A representative isolate from each species, characterized morphologically and molecularly, was tested for pathogenicity. Pathogenicity tests were performed by inoculating two plants of each *Cattleya* species. For inoculation, the isolates were subcultured onto Petri dishes containing PDA medium and incubated at 25 °C for 7 days in the dark. Healthy seedlings were obtained from a commercial orchid nursery, and two asymptomatic orchids from each *Cattleya* species were inoculated with each isolate using a 6 mm diameter disc containing mycelium from the edges of the culture, which was placed immediately into each wound previously made with a sterilized scalpel at the apex of the pseudobulb. As a control, PDA discs without the fungus were placed on the wound surface of two plants. The seedlings were kept in a humid chamber for 48 h and then transferred to a greenhouse at 28 °C. From the plants that exhibited symptoms, re-isolation was performed to recover the inoculated fungus and confirm its pathogenicity based on morphology.

## Results

A total of four isolates were obtained and morphologically identified as belonging to the *Botryosphaeriaceae* family according to [30]: one *Neoscytalidium* from *C*. *amethystoglossa*, one *Neoscytalidium* from *C*. *guttata*, and two *Lasiodiplodia* from *C*. *nobilior*.

**Fig. 2:**
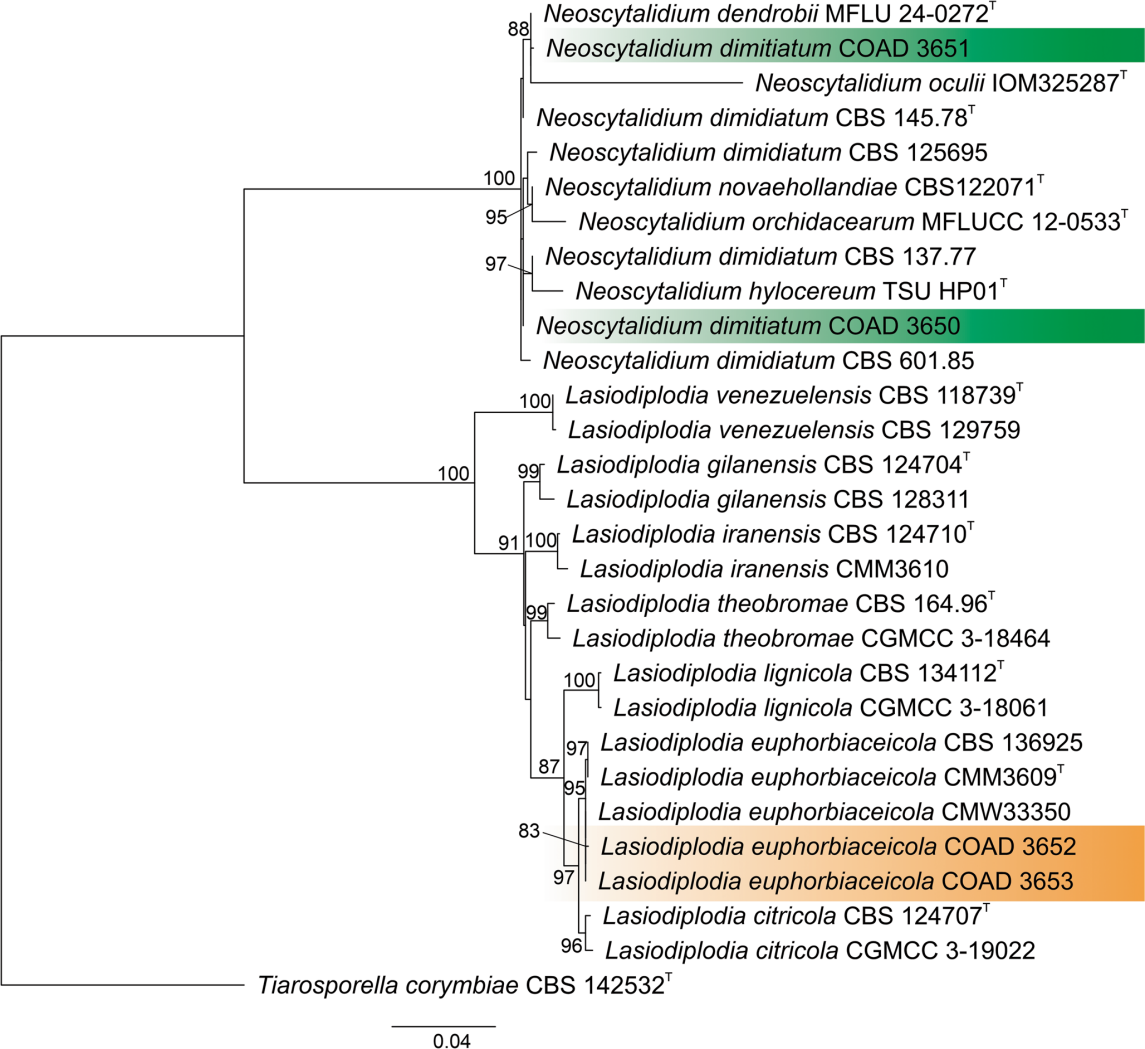
Multilocus phylogenetic tree obtained from Maximum Likehood analysis based on ITS, *TUB* and *TEF1-α* sequences. Bootstrap support values > 70% are presented. The tree was rooted with *Tiarosporella corymbiae* CBS 142,532. The isolates obtained in this study are highlighted on a colored box. The type specimens are indicated with “^T^”

**Fig. 3:**
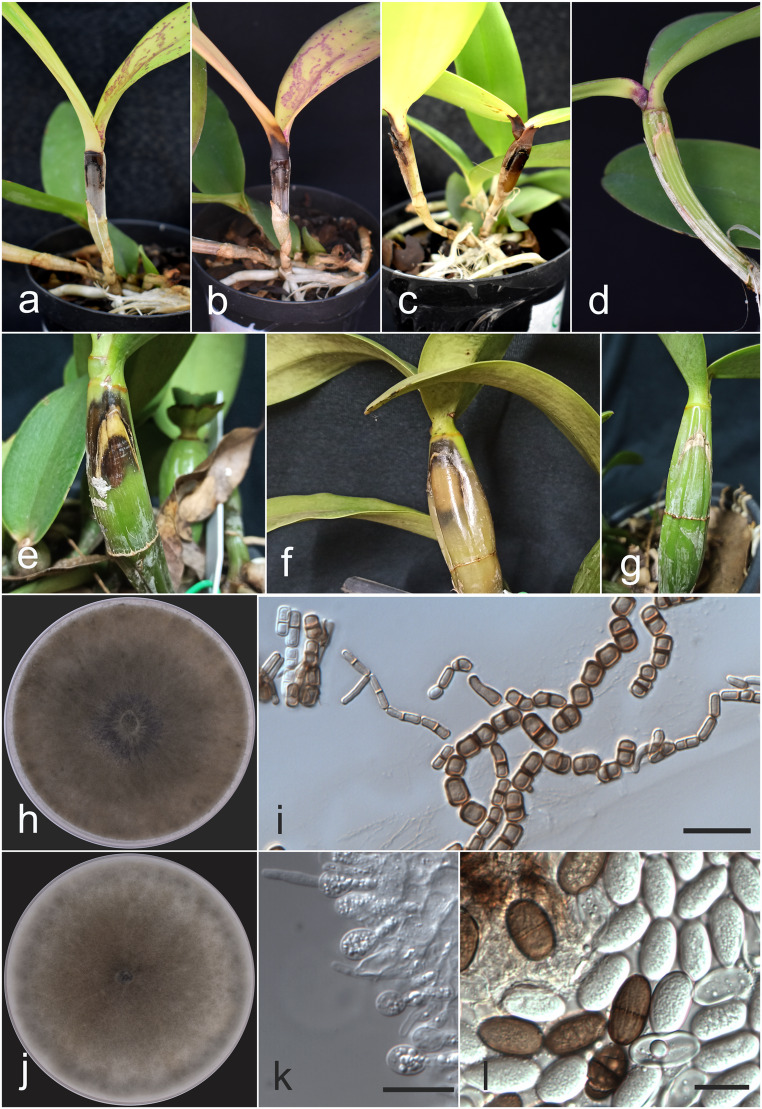
Pathogenicity test and morphological characteristics of the pathogen isolates. **a**-**b** *Cattleya amethystoglossa* inoculated with *Neoscytalidium dimidiatum* COAD 3650 after 7 and 14 days respectively. **c** *C. amethystoglossa* inoculated with isolate COAD 3651. **d** *C. amethystoglossa* (control) without symptoms after 14 days. **e**-**f** *C. nobilior* inoculated with *Lasiodiplodia euphorbiaceicola* COAD 3652 after 7 and 14 days, respectively. **g** *C. nobilior* (control) without symptoms after 14 days. **h** Colony of *N. dimidiatum* COAD 3650 on PDA after 7 days. **i** Arthroconidium of *N. dimidiatum* COAD 3650. **j** Colony of *L. euphorbiaceicola* COAD 3652 on PDA after 7 days. **k** Conidiogenous cells, immature conidia, and paraphyses of *L. euphorbiaceicola* COAD 3652. **l** Conidia of *L. euphorbiaceicola* COAD 3652. Scale bars = 20 μm

*Neoscytalidium dimidiatum* was identified based on the ML analysis of concatenated ITS, *TUB* and *TEF1*-α sequences, with our isolates clustering phylogenetically closer to *N. dendrobii* and *N. hylocerum* (Fig. 2). The isolates also retained the diagnostic morphological traits of the species, such as colonies on PDA after seven days covering the entire plate, dark brown at the center to brown at the margin, and velvety to floccose. *Conidiomata* pycnidial, partially immersed and black. *Conidiogenous cells* cylindrical to subcylindrical, hyaline, 6.1–9.4 × 3–3.5 μm. *Conidia* elliptical, when immature hyaline and aseptate, 10–11.8 × 3.8–5.2 μm, when mature biseptate with beige-brown lateral cells and a brown central cell. *Arthroconidia* cylindrical, elliptical, globose, subglobose with 0–1 septum, initially hyaline and brown when mature, 3.6–11.7 × 2.4–6.8 μm (Fig. 3). The sequences obtained and uploaded to the NCBI GenBank were the following: COAD 3650 (ITS = PX220118, TUB = PX206245, *TEF1-α* = PX216952) and COAD 3651 (ITS = PX220119, TUB = PX206246, TEF1-α = PX216953).

*Lasiodiplodia euphorbiaceicola* was identified by ML analysis of concatenated ITS, *TUB* and *TEF1*-α sequences (Fig. 2). Morphologically, *L*. *euphorbiaceicola* maintained the characteristics of the species: colonies on PDA after 7 days covering the entire plate, dark brown at the center to greyish brown at the margin, and velvety to floccose. *Conidiomata* pycnidial, superficial dark brown. *Conidiogenous cells* cylindrical to subcylindrical, hyaline, 6.7–11.1 × 3.2–5 μm. *Conidia* elliptical, when immature hyaline, truncate base and aseptate, when mature monoseptate, with thick walls, dark brown, 21.3–24.5 × 11.9–14.9 μm (Fig. 3). The sequences obtained and uploaded to the NCBI GenBank were the following: COAD 3652 (ITS = PX220120, TUB = PX206247, *TEF1-α* = PX216954) and COAD 3653 (ITS = PX220121, TUB = PX206248, *TEF1-α* = PX216955).

The pathogenicity test was repeated once using two plants per isolate, and all isolates tested were able to cause disease in their respective hosts when inoculated with a wound on the pseudobulb (Fig. 3). When the mycelial disc was inoculated without a wound, the plants did not exhibit any symptoms. The first symptoms caused by isolates of *N. dimidiatum* in *C. amethystoglossa* started four days after inoculation, whereas in isolates of *L*. *euphorbiaceicola*, symptoms were only observed seven days after inoculation. Fourteen days after inoculation of *N. dimidiatum*, necrosis of the pseudobulb was observed, followed by leaf abscission.

## Discussion

This study reveals the etiological agents of dieback in orchids, a new disease affecting commercial *Cattleya* in Brazil. On *C. amethystoglossa* and *C. guttata*, the etiological agent found was *Neoscytalidium dimidiatum*, while in *C. nobilior* was *Lasiodiplodia euphorbiaceicola*, both *Botryosphaeriaceae* species.

Despise our isolates was phylogenetically closer to *N. dendrobii* and *N. hylocerum*, in agreement with [31], who synonymized all *Neoscytalidium* species under *N. dimidiatum* owing to the absence of consistent morphological and phylogenetic distinctions, we interpret these differences as intraspecific variation. Supporting this, our isolates *N. dimidiatum* COAD 3650 and COAD 3651 differed by only four polymorphisms across 1,134 bp of concatenated alignment.

According to the orchid grower’s report, all plants that presented the disease described in this study had been transplanted shortly before the appearance of symptoms, suggesting that the disease occurs when there is an entry point for the pathogen, similar situations have also been reported for other fungi in this family [32]. *Botryosphaeriaceae* fungi have few reports of causing diseases in orchids and other herbaceous plants, being generally pathogens of woody plants [18, 33, 34]. In this group, it is also possible that the fungi remain as endophytes until some stress triggers the pathogenicity process [35], which is another possibility for the appearance of this disease. As preventive cultural control measures, transplanting should be performed under environmental conditions less favourable to the pathogen, such as low free moisture and moderate temperatures, to shorten the leaf wetness period required for conidial germination and to promote faster wound healing. Cutting tools must be disinfected between plants (e.g., using 70% ethanol, sodium hypochlorite, quaternary ammonium compounds, or flame sterilization) to prevent cross-contamination. The use of pots or transplanting systems that minimize root and tissue injuries is also recommended to reduce post-transplant stress. Since water or heat stress may trigger the transition from an endophytic to pathogenic phase, proper irrigation management and adequate shading should be adopted.

This disease may pose not only an economic threat to producers cultivating orchids, but also a conservation concern, given that *C. amethystoglossa* and *C. guttata* are endemic and classified as endangered in Brazil [36]. To the best of our knowledge, this is the first report of these species, and consequently this fungal family, causing a disease on *Cattleya* spp. in Brazil.
